# Antifungal Activity of *Copaifera langsdorffii* Desf Oleoresin against Dermatophytes

**DOI:** 10.3390/molecules181012561

**Published:** 2013-10-11

**Authors:** Danielle C. Zimmermam-Franco, Edilene B. Bolutari, Hudson C. Polonini, Antônio Márcio R. do Carmo, Maria das Graças A. M. Chaves, Nádia R. B. Raposo

**Affiliations:** 1 Núcleo de Pesquisa e Inovação em Ciências da Saúde (NUPICS), Universidade Federal de Juiz de Fora, Campus Universitário, Juiz de Fora, MG 36036-900, Brazil; E-Mails: dannyzimmermann@yahoo.com.br (D.C.Z.-F.); dibolutari@gmail.com (E.B.B.); h.c.polonini@gmail.com (H.C.P.); 2 Faculdade de Odontologia, Universidade Federal de Juiz de Fora, Campus Universitário, Juiz de Fora, MG 36036-900, Brazil; E-Mails: antoniomarcio.resende@ufjf.edu.br (A.M.R.C.); duque05@gmail.com (M.G.A.M.C.)

**Keywords:** antifungal agent, *Copaifera* spp., dermatophytosis

## Abstract

Dermatophytoses are mycoses that affect keratinized tissues in both humans and animals. The aim of this study was to investigate the antifungal activity of the oleoresin extracted from *Copaifera langsdorffii* Desf. against the strains *Microsporum canis* ATCC 32903, *Microsporum gypseum* ATCC 14683, *Trichophyton mentagrophytes* ATCC 11481 and *Trichophyton rubrum* CCT 5507. The antimicrobial activity was determined by minimum inhibitory concentration (MIC) and minimum fungicidal concentration (MFC) values. Ketoconazole and terbinafine were used as reference drugs. The copaiba oleoresin showed moderate fungicidal activity against *T. mentagrophytes* ATCC 11481 (MIC and MFC = 170 μg mL^−1^) and weak fungicidal activity against *T. rubrum* CCT 5507 (MIC = 1,360 μg mL^−1^ and MFC = 2,720 μg mL^−1^). There was no activity against *M. canis* ATCC 32903 and *M. gypseum* ATCC 14683. SEM analysis revealed physical damage and morphological alterations such as compression and hyphae clustering in the structure of the fungi exposed to the action of the oleoresin. The results stimulate the achievement of *in vivo* assays to confirm the benefits of the application of oleoresin extracted from copaiba in the treatment of dermatophytosis, both in humans and in animals.

## 1. Introduction

Dermatophytosis is a fungal disease caused in both men and animals by organisms known as dermatophytes, mainly from the genera *Microsporum* spp and *Trichophyton* spp,. These fungi use the keratin as their main substrate, and for this reason the disease affects mostly the keratinized tissues. In humans, this results in skin and scalp lesions and onychomycosis in the nails [1]. Dermatophyte infection affects approximately 40% of the world population and accounts for 30% of all cutaneous mycotic infections. In addition, the most frequent type of onychomycosis is that caused by dermatophytes, representing between 18% and 40% of all onychopathies, and about 90% of onychomycosis are caused primarily by dermatophytes, especially *Trichophyton rubrum* and *Trichophyton mentagrophytes* [2].

The current treatment against the dermatophytosis is based on antifungal drugs, mainly imidazolic derivatives and allylamines. However, these synthetic drugs are quite expensive, present adverse reactions and have a slow action. The treatment can also increase the chances of recurrence and of selecting resistant strains, in case the drugs are not properly administered [3,4,5]. These are some of the reasons that led to carry out new studies for the development of innovative antifungal products, either synthetic or natural [6,7,8].

Amongst the natural sources for antifungal agents, there is the oil or oleoresin extracted from the trees of the *Copaifera* spp genus (Leguminosae), whose popular name is copaiba [9]. It has already been studied for different ethnopharmacological activities, namely: anti-inflammatory, wound healing, antitumoral, tripanossomicidy [10], neoangiogenesis potentiating [11], periodontal disease protective [12], and antimicrobial [10,13,14] properties.

It is known that there are 72 species of *Copaifera*, but only a few reports in the literature concerning them, with the majority being related to the oil-resin of unidentified species or *C. langsdorffii*, the most widely used in folk medicine for varied purposes [15], and the reason why it was chosen in this study as a model species for the genus. In this light, the aim of this study was to investigate the *in vitro* antifungal activity of the *C. langsdorffii* oleoresin against the major filamentous fungi that cause onychomycosis, *i.e.*, *Trichophyton rubrum,* *T. mentagrophytes*, *Microsporum canis* and *M. gypseum* [2], and observe any morphological changes it causes in these fungi using scanning electron microscopy (SEM).

## 2. Results and Discussion

**Figure 1:**
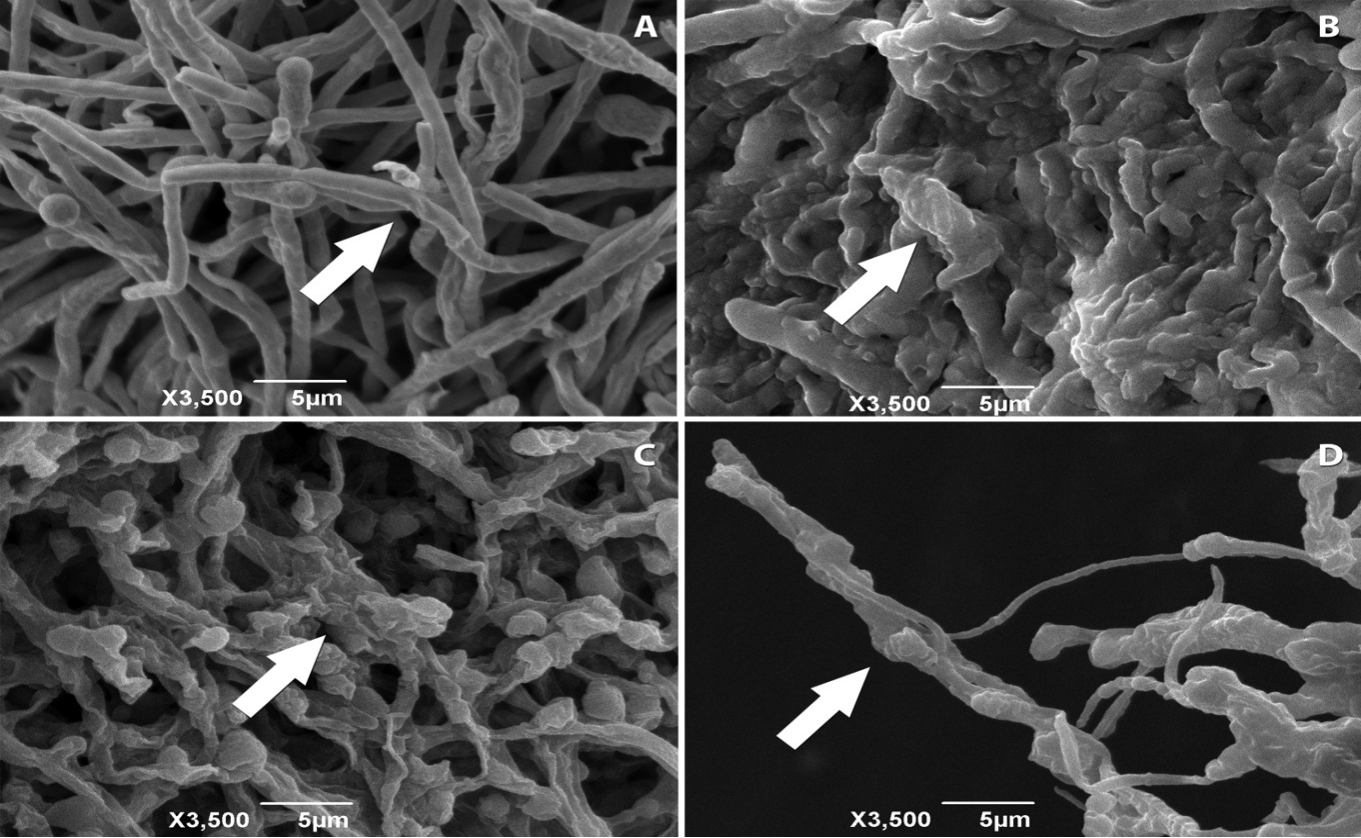
Electron micrographs of *T. mentagrophytes* ATCC 11481: (**A**) untreated—arrow indicates hyphae with morphological changes; (**B**) treated with copaiba oleoresin—arrow indicates the compression and clusters of hyphae; and (**C**) treated with ketoconazole and (**D**) treated with terbinafine—both arrows indicate the appearance of dried and wrinkled hyphae.

In order to characterize the composition of the studied oil, a gas chromatography analysis was performed, as can be seen in Table 1 and Figure 1. Peaks under 0.1% were not considered. The presence of copalic acid (1.0%), described as an originality marker, ensures that the oil comes from trees of genus *Copaifera* spp [9,16]. The major compound β-caryophyllene (31.4%), in turn, has been previously associated with antifungal activity in isolation [17,18]. Furthermore, the compounds kaurenoic acid and γ-muurolene, also present in the copaiba oleoresin studied, were reported to possess antibacterial and antifungal properties [14,19].

**Table 1:** Chemical composition of oleoresin from *C. langsdorffii* obtained by high-resolution gas chromatography.

Peak	Constituent	%
1	α-copaene	1.0
2	β-elemene	8.0
3	β-caryophyllene	31.4
4	bergamotene	10.2
5	aromadendrene	4.4
6	α-humulene	2.9
7	γ-muurolene	16.1
8	β-selinene	3.2
9	γ-cadinene	1.4
10	spathulenol	0.7
11	kaurenal	3.1
12	copalic acid	1.0
13	kaurenoic acid	0.6
14	3β-acetoxycopalic acid	0.3
Total	-	84.3

Peaks under 0.1% were not considered.

Screening of antifungal activity demonstrated the ability of copaiba oils in inhibiting only the growth of the species *T. mentagrophytes* ATCC 11481 and *T. rubrum* CCT 5507, which were then subjected to the MIC and MFC tests. For these, the *in vitro* antimicrobial activity was classified according to MIC values using the classification as follows: MIC ≤ 100 μg mL^−1^—good; 100 ≤ MIC ≤ 500 μg mL^−1^—moderate; 500 ≤ MIC ≤ 1,000 μg mL^−1^—weak; MIC ≥ 1,000 μg mL^−1^—inactive [13]. The results are shown in Table 2.

**Table 2:** Antifungal activity (minimal inhibitory concentration and minimal fungicidal concentration) of *C. langsdorfii* oleoresin and reference drugs.

Microorganism	Substances					
	*C. langsdorffii* oleoresin		Ketoconazole		Terbinafine	
	MIC (μg mL^−1^)	MFC (μg mL^−1^)	MIC (μg mL^−1^)	MFC (μg mL^−1^)	MIC (μg mL^−1^)	MFC (μg mL^−1^)
*M. canis* ATCC 32903	-	-	0.25	0.25	0.03	0.03
*M. gypseum* ATCC 14683	-	-	8.01	16.0	0.12	0.12
*T. mentagrophytes* ATCC 11481	170	170	0.25	0.25	0.03	0.03
*T. rubrum* CCT 5507	1,360	2,720	1.00	4.01	0.06	0.06

MIC: minimum inhibitory concentration. MFC: minimum fungicide concentration.

As one can see, the oleoresin showed both MIC and MFC = 170 μg mL^−1^ for *T. mentagrophytes* ATCC 11481. Towards *T. rubrum*, the MIC = 1,360 μg mL^−1^ and CFM = 2,720 μg mL^−1^ of oil. Thus, the copaiba oleoresin can be considered as a moderate inhibitor of growth of *T. mentagrophytes* ATCC 11481, and a weak inhibitor against *T. rubrum* CCT 5507. The results were similar to the results obtained by Santos *et al.* [13], who demonstrated that oleoresin from *C. langsdorffii* was also inactive against *M. canis* and *M. gypseum.* On the other hand, like in this study, it showed that different species of *Copaifera* spp did not present activity against strains of *T. mentagrophytes*.

This variation in the antifungal activity presented by oils extracted from different species of the genus *Copaifera* spp can be explained by differences in their constitution. That can occur due to genetic variations among species and among members of the same species (depending on external factors such as climate, soil, tree age, *etc.*) [20,21,22]. Therefore, one cannot generalize the antifungal activity established in this study to copaiba oils from species other than *C. langsdorffii* or even oils from the species in which the chemistry has been altered by any of the mentioned factors. For instance, a reduced production of β-caryophyllene is observed in *C. langsdorffii* specimens less exposed to attacks of lepidopterans and fungi [17,18], what can reduce the antifungal activity. However, inherent characteristics of the fungi should also be considered and investigated in order to clarify what makes them present different responses when compared to the same antifungal agents [23].

Another fact that is important to be pointed out is that the present study determined the antifungal activity of the plant as whole, and not its chemical constituents in isolation. It is known that the determination of the pharmacological activity of isolated compounds may be useful, since the use of a single substance replaces the use of the whole plant, thus allowing accurate dosing and determination of changes in the bioactivity [24]. However, it is paramount to take into account that the pharmacological activity of a medicinal plant may occur due to the synergy between its compounds [25,26,27]. Tappin [28], for example, described the copaiba oil as a case where the total chemical profile is more important than just one isolated substance to determine its pharmacological activity.

**Figure 2:**
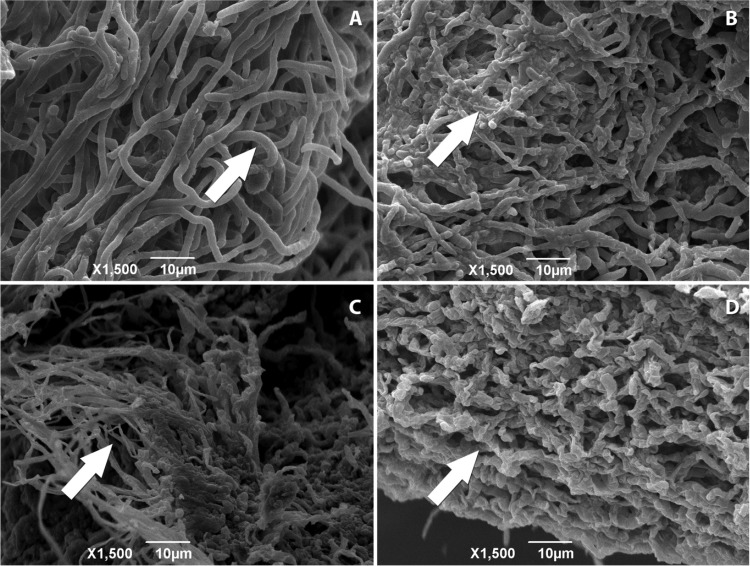
Electron micrographs of *T. rubrum* CCT 5507: (**A**) untreated—arrow indicates hyphae with morphological changes; (**B**) treated with copaiba oleoresin—arrow indicates the compression and clusters of hyphae without the original tubular shape; and (**C**) treated with ketoconazole and (**D**) treated with terbinafine—both arrows indicate the appearance of dried and wrinkled hyphae.

The MIC and MFC values were complemented by microscopy imaging. The electron micrographs obtained for *T. mentagrophytes* ATCC 11481 (Figure 1) showed differences in their structures when compared to fungi untreated with the proposed substances and those undergoing treatment with copaiba oleoresin and reference drugs. The exposure to copaiba oleoresin also caused alterations in *T. rubrum* CCT 5507 (Figure 2), which were similar to those found in *T. mentagrophytes* ATCC 11481.

The mechanism of the antifungal activity of the copaiba oleoresin is still unclear. The analysis of the electron micrographs allowed the observation that the oil promoted clustering of the structures in *T. mentagrophytes* ATCC 9533 and *T. rubrum* CCT 5507, with loss of the original tubular shape of the hyphae in both cases. The drugs ketoconazole and terbinafine, which caused changes in the fungal cell permeability with consequent modification of its structure [29,30,31,32], caused the hyphae to become parched and wrinkled. It is suggested that the copaiba oleoresin is also able to modify the permeability of fungal cells, which would imply a range of structural and biochemical changes, leading to death.

## 3. Experimental

### 3.1. Sample Characterization

A commercial sample of the oleoresin (lot CO0203) obtained by perforating the tree-trunk of *C. langsdorffii* (using an auger, which perforates the copaiba tree-trunk without damaging it) was acquired from Lazlo Aromatologia Ltda. (Belo Horizonte, MG, Brazil) and its characterization was performed on a HP5890 gas chromatograph (Hewlett-Packard, Avondale, PA, USA), equipped with a flame ionization detector. The chromatographic parameters were: BP-1 (HP) 30 m × 0.25 mm BP1 column; injection (1/50 split) of 1 µL; hydrogen as carrier gas (2 mL min^−1^); temperature of both the detector and the injector at 250 °C; and a temperature gradient (initial = 60 °C; then an increase of 3 °C min^−1^ until 220 °C) for the column. The identification of the peaks was made by calculating the retention time and comparing these with linear hydrocarbon standards C10 to C18 and literature data. Samples were diluted to 0.5% (*v/v*) in chloroform**.**

### 3.2. Microorganisms

The test organisms comprised four dermatophyte fungi: *Microsporum canis* ATCC 32903, *Microsporum gypseum* ATCC 14683, *Tricophyton mentagrophytes* ATCC 11481 (provided by the Instituto Nacional de Controle de Qualidade em Saúde da Fundação Oswaldo Cruz, Rio de Janeiro, RJ, Brazil), and *Tricophyton rubrum* CCT 5507 (kindly gifted by the Fundação André Tosello, Campinas, SP, Brazil).

### 3.3. Antifungal Screening and Susceptibility Testing

Preliminary antifungal activity was performed using the isolates of dermatophytes and oleoresin from *C. langsdorffii* at concentration of 5.44 μg mL^−1^. The assay was made as described by Souza *et al.* [33], with adaptations. Fragments (2 mm) of dermatophytes were inoculated on Sabouraud Dextrose Agar (SDA) previously incorporated with copaiba oleoresin in a sterile 24-wells plate for cell culture and the antifungal activity was determined by total growth inhibition.

The minimum inhibitory concentration (MIC) determination was performed according to National Committee for Clinical Laboratory Standards (CLSI) guidelines for filamentous fungi (document M38-A2) [34], with adaptations described in Almeida *et al.* [23]. Roswell Park Memorial Institute Medium (RPMI-1640) (Sigma–Aldrich Inc, St. Louis, MO, USA) with L-glutamine, without sodium bicarbonate and buffered with 0.165 mol·L^−1^ 3-morpholinopropanesulfonic acid (MOPS) (JT Baker, Griesheim, Germany) at pH 7.0 was used as the basal medium, either with or without antifungal agents.

Seven-day-old cultures of dermatophytes, maintained in SDA at 28 °C, were used to obtain the conidial inoculum, which was prepared by adding 8 mL of 0.9% sterile saline and 20 µL of Tween 80:DMSO (1:1, *v/v*). The densities of the suspension formed were adjusted with a spectrophotometer (Libra S12, Biochrom, Cambridge, UK), at a wavelength of 530 nm to transmittance of 68%–70%. The inocula suspensions were diluted (1:50, *v/v*) in RPMI test medium to obtain a cell number ranging from 0.4 × 10^4^–5.0 × 10^4^ colony-forming units mL^−1^. The essential oil of *C. langsdorffii* was solubilized in culture medium and evaluated at eight concentrations in the range of 0.042–5.44 μg mL^−1^. The antifungal agents (reference drugs) used in this work were ketoconazole (Janssen Cilag, Butantã, SP, Brazil) and terbinafine (Galena, Campinas, SP, Brazil), in the concentrations of 0.005–0.24 µg mL^−1^ and 0.03–16.0 µg mL^−1^, respectively. These antifungal agents were chosen because they already have protocols for testing and also because they are the most widely used in oral dosage forms in clinical practice [21,30].

The microdilution plates were incubated at 28 °C and read after 7 days of incubation. MIC was defined as the lowest concentration of oil at which the microorganism tested did not show visible growth.

The minimum fungicide concentration (MFC) was determined by means of the microdilution method [35]. Aliquots of 20 µL from the wells that did not show growth in the MIC procedure were transferred to new 96-well plates, previously prepared with 180 µL of SDA. The plates were incubated at 28 °C for 7 days. The lowest concentration with no visible growth was defined as MFC, on the plates containing broth without antifungal products.

### 3.4. Scanning Electron Microscopy

Samples for SEM observation were prepared as follows. Agar blocks treated at 28 °C for 7 days with essential oil and reference drugs (ketoconazole and terbinafine) as well as untreated control were fixed for 24 h with modified Karnovsky's fixative consisting of 2.5% (*v/v*) glutaraldehyde and 2.5% (*v/v*) paraformaldehyde in 0.05 M sodium cacodylate buffer (pH 7.2). The samples were then washed with the same buffer three times each for 10 min, and then post fixed with 1% (*w/v*) osmium tetroxide for 1 h. The post fixed samples were washed briefly with distilled water thrice and dehydrated with increasing concentrations of acetone (25%–100%, *v/v*) with an interval of 10 minutes [36]. Afterwards, the samples were transferred to a desiccator containing silica to complete the drying process. The samples obtained were assembled in aluminum stubs, with double-faced carbon tapes put on a film of aluminum foil, covered with gold in a sputter (2 mm) (FL-9496 Balzers/Furstentum Liechtenstein), and observed in a scanning electron microscope (JSM 6390LV, Jeol, Tokyo, Japan), under the conditions of 25 kW of power and work distance of 17 mm.

## 4. Conclusions

As a final consideration, one must take into account that the knowledge of copaiba’s pharmacological activity, antimicrobial in particular, has been consolidated and increased with every new study carried out with the species, the present study included. However, the species tested in the present study showed weak to moderate activity, and therefore its potential use in clinical practice is limited.
